# Tamoxifen-independent Cre-activity in *SMMHC-CreER*^*T2*^ mice

**DOI:** 10.1016/j.athplu.2022.01.002

**Published:** 2022-01-31

**Authors:** L.B. Steffensen, J. Stubbe, M. Overgaard, J.H. Larsen

**Affiliations:** a Unit of Cardiovascular and Renal Research, Department of Molecular Medicine, University of Southern Denmark, Odense, Denmark; b Department of Clinical Biochemistry and Pharmacology, Odense University Hospital, Odense, Denmark

**Keywords:** Smooth muscle cell, Atherosclerosis, Mouse, Cre-loxP

## Abstract

**Background and aims:**

Recent technological advances have established vascular smooth muscle cells (SMCs) as central players in atherosclerosis. Increasingly complex genetic mouse models have unveiled that 30–70% of cells in experimentally induced atherosclerotic lesions derive from a handful of medial SMCs, and that these can adopt a broad range of plaque cell phenotypes. Most of these models are based on the *SMMHC-CreER*^*T2*^ mouse line as Cre-driver. Importantly, Cre-activation can be controlled in time (by administration of tamoxifen, TAM), which is critical to avoid unwanted effects of premature recombination events. The aim of this study was to scrutinize an unexpected observation of TAM-independent Cre-activity in this mouse line.

**Methods:**

Cre-activity was assessed by PCR in tissues from *SMMHC-CreER*^*T2*^ mice crossed with mice homozygous for loxP-flanked (floxed) exon 4 of *Ccn2* (our gene-of-interest), and Ccn2 protein was measured in aortas by targeted mass spectrometry.

**Results:**

We observed spontaneous near-complete excision of floxed *Ccn2* in aortas from adult mice that were not treated with TAM. As a result, Ccn2 protein was significantly reduced in aortas from these mice, but not to the same extent as TAM-treated littermates. Remarkably, most of the excision was completed in 4-week-old mice. Excision was Cre-dependent, as knockout bands were negligible in heart and liver (dominated by non-SMCs) of these mice, and undetectable in the aorta in the absence of Cre.

**Conclusion:**

Our observations warrant caution, and we advocate inclusion of appropriate controls (*i.e.,* TAM-untreated mice) in future studies.

## Highlights

• Near-complete excision of a loxP-flanked gene-of-interest in *SMMHC-CreER*^*T2*^ mice that were not treated with tamoxifen.
• Tamoxifen-independent excision of loxP-flanked *Ccn2* accelerated in 4-week-old mice.
• Tamoxifen-independent Cre-activity resulted in significantly reduced aorta Ccn2 at the protein level.

## Introduction

In recent years, the use of increasingly complex transgenic mouse models has provided unprecedented insight into the role of vascular smooth muscle cells (SMCs) in atherosclerosis. Mono- and multicolor lineage tracing studies have revealed that 30–70% of cells within advanced plaques derive from a handful of clonally expanded medial SMCs, and that SMC progeny can adopt a broad range of plaque cell phenotypes (recently reviewed [[1], [2], [3]]). In addition, genetic intervention studies based on SMC-specific gene deletion have elucidated SMC-dependent mechanisms that candidate future therapeutic targeting [4,5].

**Fig. 1:**
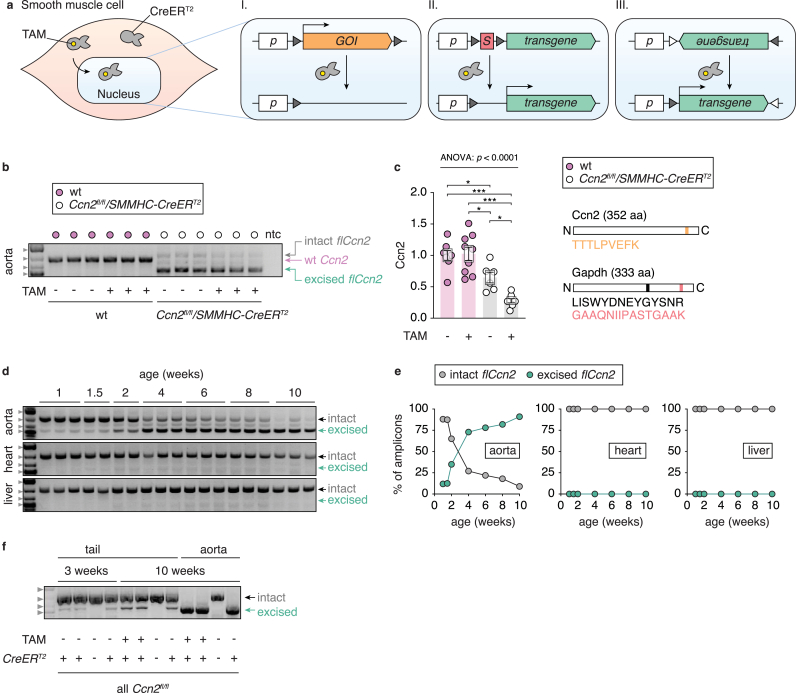
**a.** In smooth muscle cells, CreER^T2^ expression is driven by the *SMMHC* promoter and CreER^T2^ is sequestered from the nucleus in the absence of tamoxifen (TAM). Upon TAM-treatment, CreER^T2^ binds TAM and translocates to the nucleus. I-III: Three examples of CreER^T2^-loxP system use. I: Cre excises (knocks out) a floxed gene-of-interest (GOI), II: Cre excises a STOP cassette leading to transcription of a transgene (*e.g.,* a reporter gene), III: Cre inverts the open reading frame of a transgene resulting in transgene activation. loxP sites are shown as triangles. *p* = promoter; *S* = STOP cassette. **b.***Ccn2* locus genotyping of aortas from TAM-treated and -untreated wildtype (wt) and *Ccn2*^*fl/fl*^*/SMMHC-CreER*^*T2*^ males (*n* = 3 per group, randomly sampled from experiment including *n* = 6-9 mice per group). Amplicons representing intact (non-excised) *flCcn2* (1003 bp), wt *Ccn2* (878 bp), and excised *flCcn2* (587 bp) are separated by gel electrophoresis and shown on the figure. Marker bands indicated by grey arrowheads are from top: 1500 bp, 1000 bp, 700 bp, 500 bp (consistent throughout gels of Fig. 1). ntc = no template control. **c.** Targeted mass spectrometry of Ccn2 (normalized to Gapdh) in aortas from the same experiment as in Fig. 1b. Data represent mean ± SEM. ∗ = *p* ≤ 0.05, ∗ = *p* ≤ 0.001 (Tukey's multiple comparisons test following one-way ANOVA). The peptides targeted in the assay and their position in Ccn2 and Gapdh, respectively, are shown to the right. **d.***Ccn2* locus genotyping of aorta, heart, and liver from TAM-untreated *Ccn2*^*fl/fl*^/*SMMHC-CreER*^*T2*^ males at the ages indicated. **e.** Quantitative analysis of *Ccn2* locus PCRs (same samples as in Fig. 1d) separated by capillary electrophoresis. The percentages of intact and excised *flCcn2* (moles of each amplicon relative to moles of both amplicons), respectively, are shown for aorta, heart and liver. Data are shown as means. **f.***Ccn2* locus genotyping of tail biopsies from the time of unweaning (3 weeks of age), and tail biopsies and aortas from the time of sacrifice (10 weeks of age) from four mice.

To enable temporal and SMC-specific activation of transgenes (*e.g.*, a reporter fluorochrome) or inactivation of a gene-of-interest (GOI), several labs rely on the *SMMHC-CreER*^*T2*^ mouse line, developed in the Offermanns lab in collaboration with Pierre Chambon (GIE-CERBM) more than a decade ago [6] and today available from the Jackson Laboratories (B6.FVB-Tg(Myh11-cre/ERT2)1Soff/J, Stock No: 019079). In this mouse line, SMC-specific Cre recombinase (Cre) activity is ensured by placing *Cre* downstream the promoter of smooth muscle myosin heavy chain (SMMHC), which is active in fully differentiated contractile SMCs [1]. Moreover, Cre-activity is inducible since Cre is fused to mutated human estrogen receptor 1 (ER^T2^), which sequesters Cre from the nucleus until treating mice with tamoxifen (TAM, an exogenous ER agonist) (Fig. 1a). When entering the nucleus, Cre catalyzes site-specific recombination events between loxP sites. Each loxP site spans two 13 bp palindromic sequences separated by an 8 bp region, which orientation determines whether Cre will excise or invert the loxP-flanked (or “floxed”) DNA segment [7,8].

Cross-breeding the *SMMHC-CreER*^*T2*^ Cre-driver line with mice harboring a floxed GOI enables inducible SMC-specific gene knockout by Cre-mediated excision (Fig. 1a, I). Alternatively, *SMMHC-CreER*^*T2*^ mice can be used for inducible SMC-specific activation of a transgene either by excision of a STOP cassette (*i.e.,* a transcriptional roadblock) (Fig. 1a, II) or by inverting the transgene into the active orientation (Fig. 1a, III). These options (and combinations hereof) have opened a plethora of possibilities to investigate SMCs with high specificity and temporal control.

Herein, we report unexpected near-complete excision of a floxed GOI in *SMMHC-CreER*^*T2*^ mice that were not treated with TAM.

## Results

To investigate the role of a GOI (*Cellular Communication Network Factor 2* (*Ccn2*)) in vascular disease, we crossed the *SMMHC-CreER*^*T2*^ line with mice homozygous for floxed exon 4 of *Ccn2* (*Ccn2*^*fl/fl*^*/SMMHC-CreER*^*T2*^) [9].

Upon initial characterization of knockout efficiency by PCR (allowing assessment of both wildtype (wt) *Ccn2*, intact (un-excised) *flCcn2*, and excised *flCcn2* (878 bp, 1003 bp and 587 bp amplicons, respectively [9]), we noticed complete excision of *flCcn2* in aortas of 14-week-old mice that had not been treated with TAM (Fig. 1b). TAM-independent *flCcn2* excision led to a significant reduction in aorta Ccn2 at the protein level, but not to the extend observed in TAM-treated littermates (Fig. 1c).

To investigate TAM-independent *flCcn2* excision further, we analyzed aortas from 1- to 10-week-old mice that had not been exposed to TAM. We observed progressive *flCcn2* excision resulting in near-complete *Ccn2* knockout in 10-week-old mice (Fig. 1d–e). Genotyping of heart and liver samples from the same mice showed negligible *flCcn2* excision (faint bands likely represent *flCcn2* excision in the relatively small SMC population of these tissues) (Fig. 1d, two lower panels). Moreover, CreER^T2^ expression was required for *flCcn2* excision, as no excision bands were observed in tail and aorta samples from a *SMMHC-CreER*^*T2*^-negative *Ccn2*^*fl/fl*^ female littermate (the *SMMHC-CreER*^*T2*^ transgene is located on the Y-chromosome) (Fig. 1f).

## Discussion

To our knowledge, this is the first report of spontaneous Cre-activity (“Cre-leakage”) in the widely used *SMMHC-CreER*^*T2*^ line, and the first observation of spontaneous near-complete excision of a floxed GOI in any *CreER*^*T2*^ line. Our observation highlights a potential limitation to the *SMMHC-CreER*^*T2*^ line, which can be problematic depending on experimental design and the research question at hand.

The ability to induce SMC-specific gene knockout in adult mice at the onset of disease is advantageous as it minimizes the risk of affecting disease-phenotype by effects preceding disease initiation. A GOI may have known or unanticipated roles during embryology or tissue maturation in adolescent mice that could affect subsequent disease development. Likewise, untimely activation of reporter transgenes in lineage tracing studies could influence experiment outcomes leading to inaccurate conclusions.

Whether our observation is a rare case, or a widespread problem is unknown, and whether potential premature Cre-leakage has affected previous observations based on this Cre-driver is difficult to evaluate, since TAM-untreated control mice are often omitted. Our measurements of Ccn2 protein in aortas showed that spontaneous *flCcn2* excision significantly reduced Ccn2 expression, however, not to the same extend as in TAM-treated littermates (possibly due to residual intact *flCcn2* in TAM-untreated mice during the six-week period prior to sacrifice). Importantly, both TAM-treated and -untreated *Ccn2*^*fl/fl*^*/SMMHC-CreER*^*T2*^ mice had lower aorta Ccn2 compared to wt mice. This observation highlights that evaluating Cre-leakage by comparing the level of GOI expression between TAM-treated and -untreated groups can be misleading. Optimally, Cre-leakage should be evaluated either by assessing transgene activation (*e.g.*, checking if a reporter fluorochrome is detected in TAM-untreated mice) [4,10] or recombination events at the DNA level by PCR (as in this report).

The second generation *CreER*^*T2*^ system was developed to reduce leakage observed in the original *CreER* system [8], but leakage (less severe than reported here) has been reported for several *CreER*^*T2*^ lines and may be an inherent limitation of the system [[11], [12], [13], [14]]. An important determinant of recombination susceptibility is the distance between loxP sites [15,16]. A recent study demonstrated that a distance of 0.9 kb between loxP sites (similar to that in *flCcn2* mice [9]) in the *Rosa26* locus lead to 60% TAM-independent recombination, while loxP sites distanced by > 2.45 kb were not recombined [17]. Also, the genomic position of loxP sites likely affects susceptibility to Cre recombination since open chromatin is more accessible to Cre than densely packed chromatin [15]. As such, genes critical for SMC differentiation/function, which tend to be positioned in regions of open chromatin are more prone to premature excision when floxed.

Although the mechanism by which *CreER*^*T2*^ translocates to the nucleus in the absence of TAM is elusive, our observation shows that nucleus sequestration is not 100% efficient in the *SMMHC-CreER*^*T2*^ line. We notice that Cre-activity accelerates in aortas of mice between the age of 2 and 4 weeks. Whether this represents increased activity of the *SMMHC* promoter at this timepoint due to maturation (*i.e.,* final stages of differentiation) of SMCs at this time is speculative.

Going forward, we will use *SMMHC-CreER*^*T2*^ males without *flCcn2* as experimental controls and acknowledge that progressing lack of Ccn2 starting from early adolescence may contribute to any vascular phenotype observed in *Ccn2*^*fl/fl*^*/SMMHC-CreER*^*T2*^ adults.

In conclusion, we raise a potential concern to the use of the *SMMHC-CreER*^*T2*^ system. Although the severe leakage observed here may be due to a particularly susceptible flox-design, we advocate that fellow researchers perform and report appropriate control groups, *i.e.*, include TAM-untreated mice - especially during initial characterization of novel mouse lines.

## Methods

*Ccn2*^*fl/fl*^ mice (C57BL/6J) [9] (developed by Professor Andrew Leask, University of Western Ontario, and kindly provided by Professor Roel Goldschmeding, UMC Utrecht) were crossed with *SMMHC-CreER*^*T2*^ mice (B6.FVB-Tg[SMMHC-Cre/ERT2]1Soff/J, kindly provided by professor Stefan Offermanns, Max-Planck Institute for Heart and Lung Research, Germany, using the CreER^T2^ construct generated by Pierre Chambon, GIE-CERBM, France) to form breeding pairs consisting of *Ccn2*^*fl/fl*^ females and *Ccn2*^*fl/fl*^/*SMMHC-CreER*^*T2*^ males.

Data shown in Fig. 1b–c are from an experiment including TAM-treated (*n* = 6) and -untreated (*n* = 6) *Ccn2*^*fl/fl*^*/SMMHC-CreER*^*T2*^ males, and age-matched TAM-treated (*n* = 9) and -untreated (*n* = 8) wt C57BL/6J males (Taconic, Denmark). Mice were treated with TAM (T5648, Merck) (1 mg in 100 μl corn oil per day for 4 consecutive days) or corn oil at the age of 8 weeks and sacrificed at the age of 14 weeks.

Data shown in Fig. 1d–e are from all male offspring from seven litters (*n* = 19) (mice were not exposed to TAM).

Data shown in Fig. 1f are from and experiment including three *Ccn2*^*fl/fl*^*/SMMHC-CreER*^*T2*^ males and one *Ccn2*^*fl/fl*^ female littermate. Tail biopsies were harvested from 3-week-old mice (at the time of unweaning). At the age of 8 weeks, two males were treated with TAM, while one male and one female were untreated. All four mice were sacrificed at the age of 10 weeks.

Mice were euthanized by CO_2_/O_2_ inhalation, and tissues (descending thoracic aorta, heart, and liver) were snap frozen in liquid nitrogen. Tissues were lysed using DirectPCR Lysis Reagent (250-102-T, Nordic Biosite) supplemented with 0.5 mg/ml proteinase K (28229, Nordic Biosite), and used for genotyping of the *Ccn2* locus. PCR reactions (using either Taq DNA polymerase, Ampliqon, or Q5 High-Fidelity DNA Polymerase, New England Biolabs) were analyzed by agarose gel electrophoresis and capillary electrophoresis (QIAxcel Advanced System, Qiagen). Genotyping of the *Ccn2* locus was performed using primers 5′-AATACCAATGCACTTGCCTGGATGG and 5′-GAAACAGCAATTACTACAACGGGAGTGG. *SMMHC-CreER*^*T2*^ was genotyped using the standard protocol from Jackson Laboratories for this line.

Processing of snap frozen mouse aorta and quantitation of target proteins by multiple reaction monitoring (MRM) was performed as previously described [18]. Data analysis was based on peak area ratios between endogenous and heavy isotope labeled spiked peptides. Ccn2 (target peptide: TTTLPVEFK) was normalized to Gapdh (target peptides: GAAQNIIPASTGAAK and LISWYDNEYGYSNR).

## Declaration of competing interest

The authors declare that they have no known competing financial interests or personal relationships that could have appeared to influence the work reported in this paper.
